# Changes in Muscle Mass and Quality Mediate the Effect of High‐Calorie Intake on Clinical Outcomes

**DOI:** 10.1002/jcsm.70355

**Published:** 2026-08-12

**Authors:** Yassir Aarab, Fabrice Raynaud, Mathieu Capdevila, Aurelien Flatres, Gérald Hugon, Céline Lauret, Nicolas Molinari, Celia Vidal, Kada Klouche, Samir Jaber, Stefan Matecki, Boris Jung

**Affiliations:** ^1^ Saint Eloi Anesthesiology and Critical Care Medicine Montpellier University and Montpellier Teaching Hospital Montpellier France; ^2^ Intensive Care Unit Clinique Saint‐Jean Sud de France Montpellier France; ^3^ PhyMedExp University of Montpellier, INSERM U1046, CNRS UMR 9214 Montpellier France; ^4^ Biostatistics Department Montpellier University and Montpellier Teaching Hospital Montpellier France; ^5^ Medical Intensive Care Unit Montpellier University and Montpellier Teaching Hospital Montpellier France

**Keywords:** critical illness, elastography, nutrition, outcomes, skeletal muscle, ultrasound

## Abstract

**Background:**

Critically ill patients frequently experienced acquired muscle dysfunction, which adversely affects outcomes. The relationship between calorie intake, muscle dysfunction and outcomes remains unclear. This study aims to determine if early calorie intake affects patients' outcomes through changes in skeletal muscle mass or quality, and to explore changes in muscle structure and underlying molecular pathways using limb muscle biopsies in a piglet model.

**Methods:**

This translational study combines data from a prospective observational study including critically ill patients and a 72‐h ventilated piglet model with early high calorie full enteral nutrition, both recording serial ultrasound measurements of limb muscle thickness and stiffness. Patients were classified into two trajectory groups based on thickness, stiffness and calorie intake. Adjusted mediation analysis quantified the effect of calorie intake on outcomes explained by muscle trajectories. The primary endpoint was ventilator‐free days at Day 28 (VFD28). In vivo limb muscle biopsies from piglets were analysed.

**Results:**

Among 102 patients, 21% and 28% exhibited decreased thickness and stiffness by Day 7, respectively; 36% received high‐calorie intake at Day 7. Patients categorized into the ‘decreased trajectory group’ for both thickness and stiffness showed lower VFD28 (*p* < 0.01) and longer ICU stay (*p* < 0.01) compared to the ‘stable trajectory group’. High‐calorie intake was associated with significantly lower VFD28 (−8.7 days [−14.7 to −1.7]; *p* = 0.01), longer ICU stay (*p* < 0.01) and higher ICU mortality (*p* < 0.01). Decreased muscle trajectories mediated, respectively, 42% and 28% of the association between high‐calorie intake and VFD 28. The porcine model showed a limb muscle stiffness reduction associated with muscle fibre atrophy and increased intramuscular lipid content linked to decreased protein synthesis (IGF‐1 and myostatin downregulation), increased autophagy (ATG5, ATG7 and P62 increased activity) and impaired fatty acid metabolism (carnitine palmitoyltransferase 1B reduced expression).

**Conclusions:**

This study demonstrates that early calorie intake in critically ill patients adversely affects muscle mass and quality, mediating negative outcomes. The porcine model helps elucidate underlying mechanisms, with early high‐calorie intake resulting in a metabolic oversupply state associated with metabolic disorders leading to muscle fibre atrophy and lipid accumulation. These results highlight the importance of targeted nutritional strategies to preserve muscle health in critically ill patients.

## 1 Introduction

Admission to intensive care unit (ICU) and the need for organ support, notably invasive mechanical ventilation (IMV), is associated with acquired muscle dysfunction [1, 2, 3]. ICU‐acquired muscle weakness occurs in 25%–75% of critically ill patients [3, 4, 5]. It correlates with increased mortality, extended hospital days and impaired recovery, primarily stemming from muscle mass loss secondary to increased protein catabolism and decreased protein synthesis [4, 5, 6, 7]. However, beyond mass, critical illness also leads to functional impairments related to decreased muscle quality.

The acute phase of critical illness is characterized by increased protein catabolism and significant muscle wasting [2, 4, 6, 7]. Optimal nutritional support during this phase is crucial, yet it remains a contentious topic [8, 9]. Unintended low‐calorie intake is linked to unfavourable outcomes (higher mortality and longer length of stay), whereas intentional trophic feeding lead to similar outcomes when compared to a high‐calorie intake strategy [9, 10]. Intentional high‐calorie intake during the acute phase of critical illness fails to improve outcomes and is associated with greater muscle waste and weakness [9, 11, 12]. Several mechanisms may explain the lack of benefit of high‐calorie intake, including anabolic resistance (when muscle wasting cannot be counteracted by providing macronutrients), which results in overfeeding illustrated by hyperglycaemia, hypertriglyceridemia and liver dysfunction, and impaired repair pathways such as autophagy [9]. The randomized multicentre NUTRIREA‐3 trial found similar mortality rates between calorie restriction and high‐calorie intake but demonstrated lower complication rates and faster recovery in the calorie restriction group [13].

This translational study aims to explore the association between early calorie intake, limb muscle ultrasound parameters and ventilator‐free days at Day 28 (VFD28), ICU length of stay and mortality. Additionally, this study explored the association between muscle ultrasound parameters with muscle structure and cellular pathways involved in muscle homeostasis using limb muscle biopsies in a mechanically ventilated piglet model receiving early full enteral high‐calorie intake.

## 2 Methods

### 2.1 Study Population and Setting

This prospective observational study was conducted in the medical ICU at Montpellier University Hospital, France, from December 2017 to June 2018. All consecutive adult patients admitted with at least one organ failure, as defined by the Sequential Organ Failure Assessment (SOFA) score with an expected stay of at least 3 days were eligible and enrolled within 24 h of admission. Noninclusion criteria encompassed pregnancy, pre‐existing neuromuscular disease, spinal cord injury, intracranial disease, transfer from another ICU, age under 18 and refusal to participate. Patients were enrolled within 24 h of admission regardless of the need for IMV. In accordance with French law, written consent was waived as the present study formed part of an image databank. Institutional Review Board approval for the initial study protocol (2017‐CLER‐MTP‐09‐16) was obtained, with subsequent approval secured for the inclusion of additional data related to nutritional interventions (2025‐11‐368).

### 2.2 Collected Data

Demographic characteristics were recorded at ICU admission. All clinical variables were prospectively collected daily from admission until death or hospital discharge. Calorie and macronutrient intake (protein, carbohydrate and lipid intake from enteral/parenteral nutrition and glucose infusions) were collected daily. Nutritional and non‐nutritional intakes were combined to calculate total energy in kilocalories (kcal/kg/day) and protein loads (g/kg/day) of actual bodyweight.

Right biceps brachii (Bb) measurements of thickness and stiffness using ultrasound B‐mode and shear wave elastography (SWE) were performed in triplicate at Day 0 then every other day until ICU discharge, death or Day 28, whichever came first (Figure S1). Images were analysed offline by two blinded operators to ensure reproducibility (see Supporting Information) [14]. Ultrasound muscle thickness may increase or decrease according to the combination of changes in all its components including the muscle fibres but also connective tissue, the vessels, inflammatory or lipid accumulation and muscle oedema. SWE gives a spatial representation of muscle stiffness and provides measures of muscle quality [15, 16]. Increased muscle stiffness is associated with muscle fibrosis, and decreased muscle stiffness is consistent with the development of muscle injury and weakness, which reflect muscle atrophy and fat infiltration [15, 16].

### 2.3 Nutritional Support

Artificial nutrition was initiated within 24 h from ICU admission or after hemodynamic stabilization, prioritizing enteral nutrition. Parenteral nutrition was administered only if enteral was contraindicated or insufficient. Energy requirements were estimated using the Harris and Benedict equation [8]. Protein targets ranged from 1.2 to 1.5 g/kg of pre‐admission body weight daily without renal adjustment.

### 2.4 Definitions

We defined the acute phase as the first 7 days after ICU admission. We used a 25‐kcal/kg/day threshold at Day 7 in line with contemporary guidelines for calorie targets [8]. Early high and standard calorie intake were, respectively, defined as higher and lower than 25 kcal/kg/day during the acute phase. Trajectory groups for calorie intake were modelled using the 25‐kcal/kg/day threshold during the acute phase (Day 7) to define the ‘early high‐intake’ and ‘standard‐intake’ groups.

Trajectory groups of Bb thickness and stiffness over time were defined either as ‘decreased’: decrease by more than 10% from baseline at Day 7 or ‘stable’: change by less than 10% from baseline at Day 7.

Malnutrition was defined according to the ESPEN consensus criteria (BMI < 18.5 kg/m^2^ or unintentional weight loss > 10% indefinite or > 5% over 3 months combined with low BMI) [8].

### 2.5 Mechanically Ventilated Piglet Model and Muscle Biopsies Analysis

An experimental study involving 12 healthy piglets (25–35 kg) was conducted after ethical approvement (APAFIS No. 15866‐2018070509081002), to explore muscle structural and metabolic changes associated with early high‐calorie nutrition. We aimed to mimic a baseline healthy state (D0) and the effect of an early phase of basic ICU interventions: sedation, immobilization, IMV and nutrition (D3). Piglets underwent volume‐controlled ventilation for 72 h, receiving immediate enteral nutrition at 30 kcal/kg/day and 1.2‐g/kg/day protein infusion, according to ICU early high‐calorie nutritional strategy [16]. Ultrasound images for cross‐sectional area (CSA) and stiffness measurements of limb muscle were recorded at Day 0 (D0) and Day 3 (D3) (Figure S2). Limb muscle in vivo biopsies were harvested at baseline immediately after intubation (D0) and prior to euthanasia on D3 for 10 piglets. Briefly, we evaluated muscle fibre CSA, cytochrome C oxidase (Cox) staining intensity, lipid accumulation (Oil red O staining) and transcripts level of key regulators involved in protein synthesis and breakdown including insulin‐like growth factor 1 (IGF‐1), lipid metabolism using carnitine palmitoyl transferase‐1 (CPT1b) and mitochondrial oxidative capacity (PGC‐1α). The detailed study protocol is provided in the electronic supplement.

### 2.6 Endpoints

The primary outcome was VFD28. Secondary outcomes were ICU length of stay and mortality. Exposure was calorie intake. Potential mediators were trajectories of Bb thickness and stiffness over time.

The exploratory outcomes in piglet model were to describe the changes between D0 and D3 and the association between muscle mass and quality evaluated by ultrasound, histological muscle structure and muscular expression of key factors implicated in protein homeostasis, lipid metabolism and mitochondrial energy production capacity. Based on our previous work on diaphragm, we used a 10% cut‐off change in stiffness as in critically ill patients to compare piglets with a decrease of 10% or more in stiffness (decreased) and piglets with a change of less than 10% (stable) [16].

### 2.7 Statistical Analysis

Descriptive statistics were used to summarize baseline characteristics, with comparisons performed using appropriate tests (chi‐square, *t*‐test or Wilcoxon). Associations with outcomes were assessed via univariate and multivariable linear regression models, with Cox proportional hazards for mortality. Trajectories of muscle ultrasound parameters over time were modelled using group‐based multivariate trajectory analysis. Causal mediation analysis with the R mediation package quantified whether muscle property changes mediated the impact of calorie intake on outcomes, based on 5000 bootstrap iterations (Figure S3).

All analyses were adjusted for confounders: age, sex, SOFA score and SAPS II score at admission, sepsis, septic shock, neuromuscular blockade, corticosteroids and dialysis. All tests were two‐sided, with *p* < 0.05 deemed significant. Analyses were performed using R version 4.4.0.

## 3 Results

### 3.1 Demographics and Ultrasound Measurements at Baseline

**FIGURE 1:**
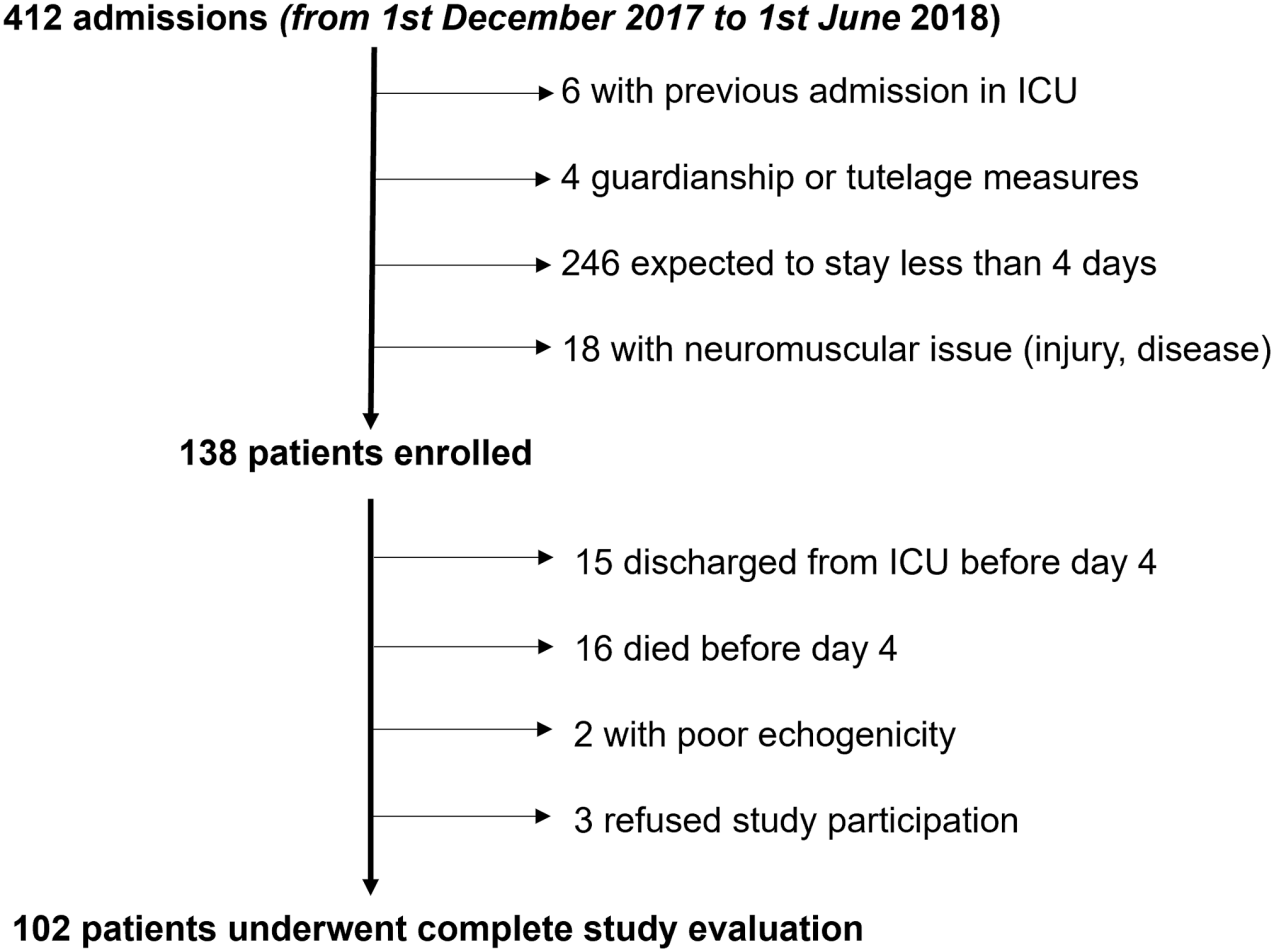
Study flow chart of critically ill patients. Flow chart of patients admitted to our unit during the study. Consecutive patients with at least one organ failure and an expected duration of ICU stay equal to or greater than 3 days were enrolled. Of 412 adults, 138 were enrolled, 36 were excluded and 102 completed the study evaluation. Abbreviation: ICU, intensive care unit.

Between December 2017 and June 2018, 102 critically ill patients were enrolled in the study, each undergoing a minimum of three ultrasound evaluations (Figure 1). Demographic and clinical characteristics are reported in Table 1. At Day 0, 67 patients (71%) received IMV, and finally, 88 patients (86%) were mechanically ventilated during the ICU stay. The duration of IMV was 6 [4, 5, 6, 7, 8, 9, 10, 11, 12, 13, 14, 15] days with 2.8 [1.1–5.7] days in controlled mode. Mean SAPS II score was 59 ± 19, and 23 patients (23%) presented malnutrition on admission.

**TABLE 1:** Demographics, clinical characteristics and outcomes of the study population according to calorie intake groups.

Characteristics	Study population (*n* = 102)	Daily caloric intake during the first week		
		High intake (*n* = 37)	Standard‐intake (*n* = 65)	*p*
Demographics				
Age	61 (17)	61 (18)	61 (16)	0.85
Sex (male)	74 (73)	28 (76)	46 (71)	0.59
Body mass index (kg/m^2^)	25 (±5)	24 (±5)	25 (±6)	0.85
SAPS II	59 (±19)	62 (±20)	57 (±18)	0.24
SOFA score at admission	8 [6–11]	7 [5–11]	8 [6–10]	0.61
Comorbidities				
COPD	18 (18)	8 (22)	10 (15)	0.43
Ischemic heart disease	14 (14)	4 (11)	10 (15)	0.52
Chronic heart failure	10 (10)	2 (5)	8 (12)	0.32
Diabetes mellitus	24 (24)	6 (16)	18 (28)	0.19
Chronic kidney disease	15 (15)	7 (19)	8 (12)	0.37
Cancer	34 (33)	14 (38)	20 (31)	0.47
Cirrhosis	5 (5)	2 (5)	3 (5)	0.86
Inflammatory disease	8 (8)	4 (11)	4 (6)	0.46
Chronic corticosteroid treatment	22 (22)	10 (27)	12 (18)	0.31
Malnutrition	23 (23)	10 (27)	13 (20)	0.41
Sepsis‐3 criteria within first week	80 (78)	27 (73)	53 (82)	0.31
Septic shock within first week	45 (44)	14 (38)	31 (48)	0.34
Number of organ failures at admission	3 [2–3]	3 [2–4]	3 [2–3]	0.31
Primary reason for admission				
Acute respiratory failure	56 (55)	20 (54)	36 (55)	0.90
Cardiac arrest	10 (10)	4 (11)	6 (10)	0.99
Acute heart failure	3 (3)	0 (0)	3 (5)	0.55
Coma—encephalopathy	10 (10)	4 (14)	5 (8)	0.49
Sepsis	15 (15)	4 (11)	11 (17)	0.40
Other	8 (8)	4 (11)	4 (6)	0.46
Clinical management during ICU stay				
Use of aminoglycosides	34 (33)	10 (27)	24 (37)	0.31
Use of corticosteroids	56 (55)	21 (57)	35 (54)	0.78
Use of sedatives	88 (86)	34 (92)	54 (83)	0.21
Use of NMBA	61 (60)	28 (76)	33 (51)	0.01
Use of invasive MV	88 (86)	34 (92)	54 (83)	0.34
Duration of invasive MV^b^	8 [5–16]	14 [9–24]	6 [4–10]	< 0.01
Need for vasopressors	88 (86)	32 (86)	56 (86)	> 0.99
Duration of vasopressor infusion^b^	4 [3–7]	5 [3–10]	4 [3–6]	0.21
Need for dialysis	27 (26)	11 (30)	16 (25)	0.57
Outcomes				
Prolonged or difficult weaning^a^	37 (36)	19 (51)	18 (28)	0.02
Prolonged or difficult weaning^b^	37 (43)	19 (59)	18 (33)	0.02
Ventilator free days to Day 28^a^ ^c^	21 [11–25]	7 [4–17]	23 [19–26]	< 0.01
Ventilator free days to Day 28^b^	19 [4–24]	4 [0–15]	23 [18–25]	< 0.01
ICU length of stay^a^	12 [7–19]	17 [12–28]	10 [6–16]	< 0.01
ICU mortality^a^	23 (23)	16 (43)	7 (11)	< 0.01
Day 28 mortality^a^	19 (19)	13 (35)	6 (9)	< 0.01

*Note:* All data are presented as either *n* (%) for categorical variables, mean (±standard deviation) or median [interquartile] for continuous variables, as appropriate.

Abbreviations: CMV = controlled mechanical ventilation; COPD = chronic obstructive pulmonary disease; ICU = intensive care unit; MV = mechanical ventilation; NMBA = neuromuscular blockade; SAPS II = Simplified Acute Physiology Score; SOFA = Severity of Organ Failure Assessment.

^a^ Results are presented for the total study population (102 patients).

^b^ Results are presented for patients who received the mentioned therapy.

^c^ Durations are expressed in days.

We collected 664 and 652 valid ultrasound measurements for thickness and stiffness, respectively, after excluding 26 and 38 images for poor quality criteria (Figure S1). Upon ICU admission, Bb thickness was 2.2 ± 0.4 cm and stiffness was 25.2 ± 7.0 kPa. No correlation was found between muscle thickness and stiffness as absolute values; however, percentages of variation from baseline were significantly correlated (Figure S4).

Nutrition was started on Day 1 in 45 patients (44%) for enteral route, and in 12/102 (12%) patients for parenteral. The provision of calories and proteins increased over the first week from a median intake of 5.6 [1.1–13.2] kcal/kg and 0.2 [0.1–0.5] g/kg of protein on Day 1, to 23.4 [13–27.0] kcal/kg and 1.0 [0.4–1.3] g/kg of protein on Day 7 (Table S1).

### 3.2 Trajectories of Bb Thickness, Bb Stiffness and Calorie Intake Over Time

**FIGURE 2:**
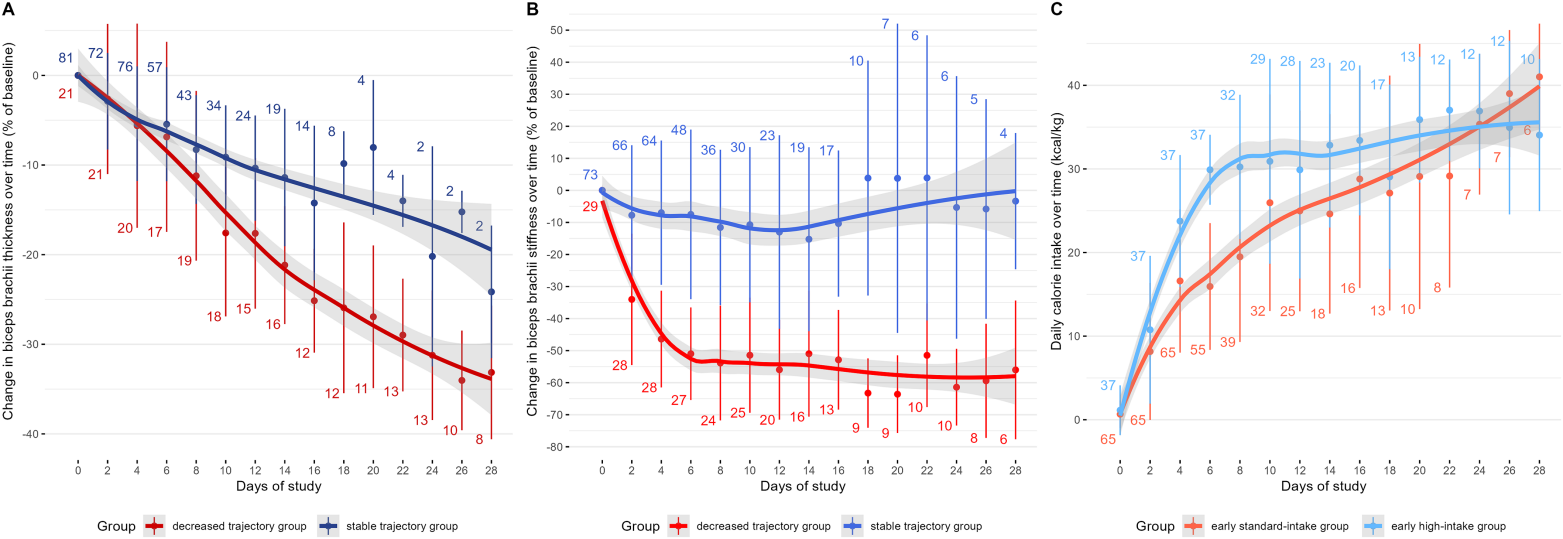
Trajectories of biceps brachii thickness (A), biceps brachii stiffness (B) and daily calorie intake (C) over time in critically ill patients. Subjects are categorized into two trajectory groups according to the magnitude and direction of changes from baseline to the final measurement during the first week of the ICU stay. For thickness and stiffness, ‘decreased trajectory group’ and ‘stable trajectory group’ were formed using a group‐based multivariate trajectory modelling approach. Percentage of change from baseline (enrolment day) is represented on the y‐axis, days of follow‐up on the x‐axis. For calorie intake, a threshold of 25 kcal/kg/day on Day 7 was used for ‘standard‐intake group’ and ‘high‐intake group’. Daily calorie intake is represented on the y‐axis, days of follow‐up on the x‐axis. Mean and standard deviation are plotted for each group on each study day. Numbers shown next to each data point indicate the number of measurements on each study day. Shared area indicates confidence intervals. Trend line and confidence intervals (shaded area) were fitted by Loess smoothing.

Overall change over time in Bb thickness showed a ‘decreased trajectory group’ for 21 patients (21%) defined as a decrease by more than 10% at Day 7, exceeding 30% at Day 28 (Figure S9). The ‘stable trajectory group’ showed a decrease by less than 10% at Day 7, followed by a gradual decline reaching a 20% decrease at Day 28 (Figure 2A). Similarly, Bb stiffness showed a ‘decreased trajectory group’ for 29 patients (28%) with a rapid decrease reaching 50% from baseline at Day 7 and continued to decline beyond 60% at Day 28. The ‘stable trajectory group’ experienced minimal decrease by less than 10% at Day 7 (Figure 2B).

Calorie intake trajectories grouped patients into a ‘standard‐intake group’ (*n* = 65, 64%) who progressively increased intake to 16.0 [7.1–21.9] kcal/kg at Day 7, and an ‘early high‐intake group’ (*n* = 37, 36%) characterized by rapid achievement of 28.6 [26.6–32.4] kcal/kg at Day 7 (Figure 2C and Table 1).

Both thickness and stiffness ‘decreased trajectory group’ showed lower VFD28 (*p* < 0.01) and longer ICU length of stay (*p* < 0.01) when compared to the ‘stable trajectory group’ (Table S2). The ‘early high‐intake group’ had lower VFD28 (*p* < 0.01), longer ICU length of stay (*p* < 0.01) and higher ICU mortality (*p* < 0.01) when compared to the ‘standard‐intake group’ (Table 1). In adjusted multivariate analysis, factors significantly associated to lower VFD28 were higher SAPSII score, ‘decreased thickness trajectory group’ and ‘high‐intake group’ (Table S3).

### 3.3 Mediation Analysis: Muscle Thickness and Stiffness Trajectories as Mediators of Clinical Outcomes Associated to Early High‐Calorie Intake

The total effect of an ‘early high‐calorie intake’ on VFD28 was significatively mediated by Bb thickness trajectory (*p* = 0.04) and Bb stiffness trajectory (*p* = 0.01). The proportions of these effects were 42% and 28%, respectively (Table 2, Figures S5 and S6).

**TABLE 2:** Direct and indirect effect of early high‐calorie intake on outcomes—adjusted mediation analysis.

Outcome	Mediator	Total effect	Direct effect	Indirect effect	Proportion of mediation	*p*
VFD 28 (days)	Bb thickness trajectory	−8.7 [−14.7–1.7]	−5.0 [−10.1–0.8]	−3.7 [−7.8–0.2]	42%	0.04
	Bb stiffness trajectory	−8.9 [−13.7–4.7]	−6.5 [−11.3–1.0]	−2.5 [−6.6–0.4]	28%	0.01
ICU stay (days)	Bb thickness trajectory	7.7 [1.8–16.5]	4.8 [0.2–9.7]	2.9 [0.2–9.11]	38%	0.03
	Bb stiffness trajectory	8.6 [1.9–16.0]	7.33 [0.9–14.4]	1.24 [−0.4–3.7]	14%	0.19

*Note:* Adjusted on age, sex, SOFA score, SAPSII score, sepsis, septic shock, use of NMBA, use of corticosteroids and need for dialysis.

Abbreviations: Bb = biceps brachii; ICU = intensive care unit; VFD28 = ventilatory‐free days at Day 28.

The total effect of an ‘early high‐intake’ on ICU stay was significatively mediated by Bb thickness trajectory (*p* = 0.03) but not by Bb stiffness trajectory (*p* = 0.19). The proportion of this effect was 38% (Table 2, Figures S7 and S8). All steps of the mediation analysis are detailed in Table S4.

### 3.4 Piglet Model Relation Between Muscle Thickness and Stiffness, Muscle Fibre CSA, Lipid Accumulation and Protein Homeostasis

**FIGURE 3:**
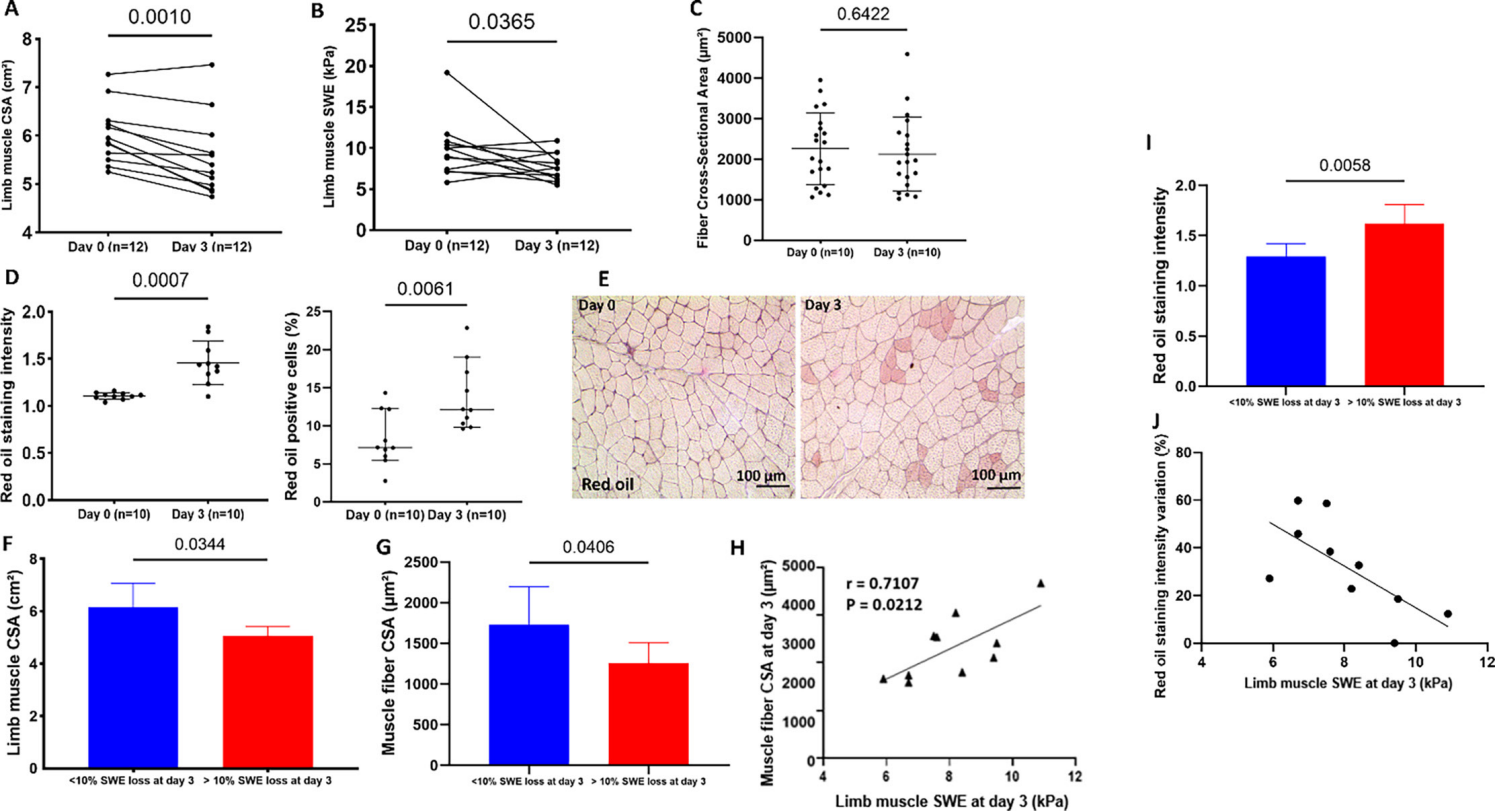
Changes in limb muscle mass, quality, protein and lipid homeostasis, obtained in a 3‐day model of mechanically ventilated piglets with high‐calorie intake. (A) Quantitative analyses of the limb muscle cross‐sectional area (CSA) between Day 0 and Day 3. (B) Quantitative analyses of the limb muscle stiffness (SWE) between Day 0 and Day 3. (C) Quantitative analysis of muscle mass using muscle fibre (Type 1 and 2) CSA between Day 0 and Day 3. (D) Quantitative analysis of global intramuscular lipid content (muscle quality) using Oil red O staining intensity with arbitrary units (left panel) and percentage of red oil positive cells (right panel) between Day 0 and Day 3. (E) Representative Oil red O staining of limb muscle cryo‐sections at Day 0 and Day 3. (F) Limb muscle CSA according to decrease in stiffness (SWE) at Day 3. (G) Muscle fibre CSA according to decrease in stiffness (SWE) at Day 3. (H) Relationship between muscle fibre CSA at Day 3 (ordinate) and limb muscle stiffness (SWE in abscissa) at Day 3. (I) Oil red O staining intensity according to decrease in stiffness (SWE) at Day 3. (J) Relationship between variation of Oil red O staining intensity as a percentage from baseline (ordinate) and limb muscle stiffness (SWE in abscissa) at Day 3. [Correction added on 25 August 2026, after first online publication: Panel F in Figure 3 has been corrected in this version.]

Using ultrasound, limb muscle CSA and stiffness significantly decreased between D0 and D3 [6.1 ± 2.4 cm vs. 5.6 ± 2.3 cm (*p* < 0.01)] and [9.8 ± 3.4 kPa vs. 7.7 ± 1.6 kPa (*p* = 0.04)], respectively (Figure 3A,B). When using the 10% cut‐off change, stiffness remained stable in six piglets (five with limb muscle biopsies) and decreased in six piglets.

On histology, Type I and Type II fibres CSA showed no significant changes in muscle mass between D0 and D3 (Figure 3C). Oil red O staining showed that intramuscular lipid accumulation increased between D0 and D3 (Figure 3D,E). A decrease in stiffness by more than 10% during the 3‐day experiment was associated with smaller limb muscle CSA (ultrasound, Figure 3F), greater reduction in Type I and Type II fibres CSA (histological atrophy, Figure 3H) and increased intramuscular lipid content (Figure 3I,J).

**FIGURE 4:**
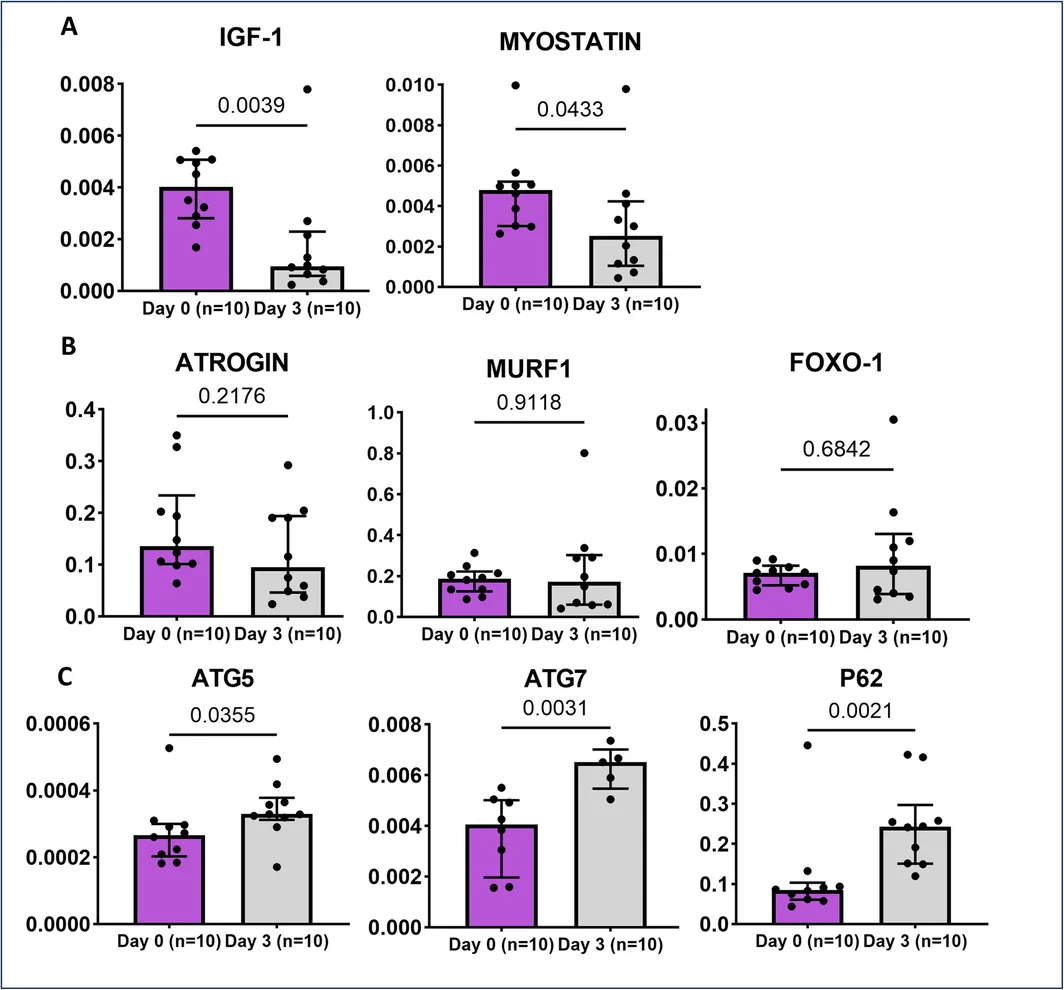
Changes in muscle protein and lipid homeostasis, obtained in a 3‐day model of mechanically ventilated piglets with high‐calorie intake. (A) Quantitative analysis between Day 0 and Day 3 of muscular expression levels obtained by Rt‐PCR (arbitrary unit) of insulin‐like growth factor 1 (IGF‐1; left panel) and myostatin (right panel). (B) Quantitative analysis between Day 0 and Day 3 of muscular expression levels obtained by Rt‐PCR (arbitrary unit) of Atrogin, Muscle Ring Finger 1 (MuRF1) and FOXO‐1. (C) Quantitative analysis between Day 0 and Day 3 of muscular expression levels (obtained by Rt‐PCR, arbitrary unit) of autophagy protein 5 (ATG5), autophagy protein 7 (ATG7) and P62.

We also investigated the modification of key expression factors involved in protein metabolism and muscle mass homeostasis. We found a downregulation of IGF‐1, which is implicated in protein synthesis associated to a decreased expression levels of myostatin, a protein that negatively regulates skeletal muscle growth (Figure 4A), no significant alterations in the expression levels of Atrogin, Muscle Ring Finger 1 (MuRF1) and FOXO‐1 indicating a stable proteolytic activity of the proteasome system (Figure 4B), and an expression of proteins involved at different stages of the autophagic pathway including Autophagy protein 5 (ATG5), Autophagy protein 7 (ATG7) and P62 (Figure 4C).

**FIGURE 5:**
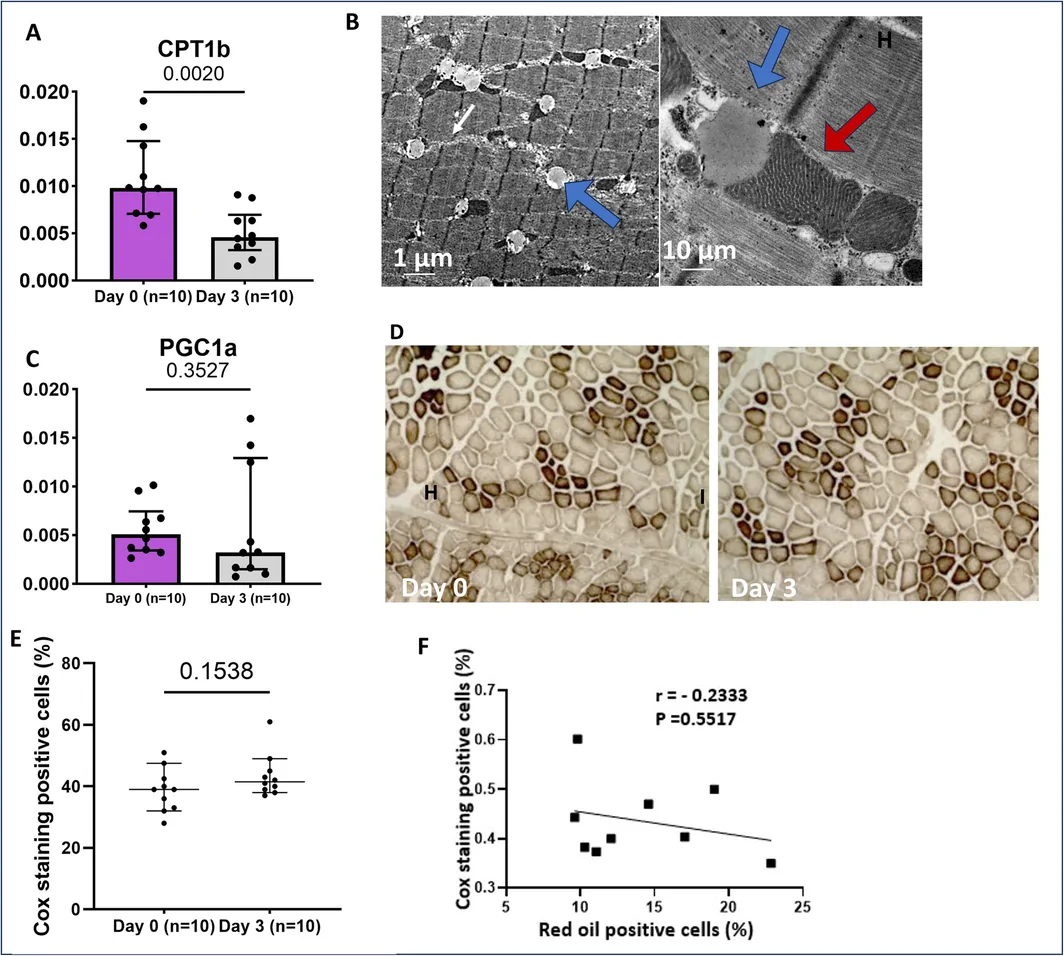
Changes in muscle lipid transport and metabolic capacity, obtained in a 72‐h model of mechanically ventilated piglets with high‐calorie intake. (A) Quantitative analysis between Day 0 and Day 3 of muscular expression levels obtained by Rt‐PCR (arbitrary unit) of carnitine palmitoyl transferase‐1b (CPT1b), a transporter of fatty acids into mitochondria. (B) Representative electron microscopy images of longitudinal ultrathin section obtained from limb muscle at Day 3 with lipid droplets accumulation (blue arrows) near mitochondria without any morphological alteration (red arrow). (C) Quantitative analysis between Day 0 and Day 3 of muscular expression levels obtained by Rt‐PCR (arbitrary unit) of PGC‐1α, a key regulator of mitochondrial biogenesis. (D) Representative cryo‐section images with Cox staining at Day 0 (left) and Day 3 (right). (E) Quantitative analysis between Day 0 and Day 3 of percentage of Cox staining positive cells. (F) Correlation between lipid content (percentage of Oil red O positive cells, abscissa) and oxidative capacity (Cox staining positive cells, ordinate) at Day 3.

Regarding lipid metabolism, in accordance to increased intramuscular lipid accumulation at D3 compared to D0 (Figure 3D,E), we observed a downregulation of muscle CPT1b, a transporter of fatty acids into mitochondria associated with lipid droplet accumulation near mitochondria highlighted by electron microscopy (Figure 5A,B).

We did not observe any morphological alterations in the mitochondrial ultrastructure with similar mitochondrial density between D0 and D3 attested by the stable expression levels of PGC‐1α (Figure 5C). We observed a stable level of cytochrome C oxidase (Cox) staining indicating a constant muscular oxidative metabolic capacity between D0 and D3 (Figure 5D,E). The absence of correlation between the number of Oil red O positive cells and the number of COX‐positive cells suggests that lipid accumulation is independent of mitochondrial oxidative capacity (Figure 5E).

## 4 Discussion

This study highlights key associations between early high‐calorie intake at the acute phase in critically ill patients and poor clinical outcomes, which are partially mediated by impaired skeletal muscle mass (thickness) and quality (stiffness). Additionally, our 72‐h mechanically ventilated piglet confirms that nutritional oversupply results in metabolic disorders encompassing impaired protein synthesis and accelerated autophagy along with conserved mitochondrial oxidative metabolic capacity and impaired fatty acids transport to the mitochondria, leading to accumulation of intramuscular lipid accumulation and muscle fibre atrophy, which are both associated with decreased muscle stiffness as measured by SWE.

Muscle dysfunction occurs due to factors such as immobilization, inflammation, ageing and malnutrition [4, 6, 17, 18]. During critical illness, excessive catabolism exacerbates muscle dysfunction [2, 7]. This catabolic state is typically observed in the acute phase of critical illness [6, 19]. Greater calorie deficit has been associated with unfavourable outcomes. Considering that prolonged calorie deficits could contribute to catabolism, nutritional strategies involving the need for sufficient calorie intake have been advocated. However, recent trials have challenged that assumption, and some have shown potential harm with early high‐calorie intake during the acute phase [9, 12, 13]. Evidence for causality between calorie intake, loss of muscle mass and clinical outcomes is still lacking [9]. Our mediation analysis suggests a potential causal relation between ‘high‐calorie intake’ at the acute phase of critical illness and poor clinical outcomes (VFD28 and ICU stay), which is mediated by decline in muscle mass (thickness) and quality (stiffness).

To assess muscle mass and quality, we used B‐mode ultrasonography to measure thickness and SWE to measure stiffness of the Bb [2, 20]. We chose to measure Bb instead of quadriceps because its measurement is more reliable for both mass and quality assessment [14, 15, 21]. Atrophy of Bb is also associated with in‐hospital mortality and physical function impairments in mechanically ventilated critically ill patients [22]. We herein report two distinct thickness and stiffness trajectories during the acute phase of ICU stay (Figure 2). The plateau in muscle stiffness observed after the initial decline may be explained by a transition from early qualitative alterations in muscle tissue (e.g., lipid accumulation, oedema and disruption of myofibrillar architecture) that occur rapidly during the acute phase, to a new ‘low‐stiffness’ steady state reflecting viscoelastic properties of the remaining connective tissue, which limits further change in elastic modulus. In contrast, loss of muscle mass, as reflected by thickness, likely progresses more slowly and continuously through sustained protein catabolism and reduced synthesis. Decrease in thickness was associated with higher length of ICU stay and less VFD28 consistent with existing knowledge that identifies muscle mass loss as a negative contributor to clinical outcomes [2, 4, 5, 17, 22]. We also show that decrease in limb muscle stiffness over time in critically ill patients is associated with higher length of ICU stay and less VFD28. This is the first report of an association between outcomes and a non‐invasive and non‐volitional measurement of limb muscle quality. Although we did not perform quantitative echo intensity analysis in our clinical cohort, the piglet model showed that decreased stiffness was associated with both muscle fibre atrophy and increased intramuscular lipid content, supporting the interpretation of SWE changes as reflecting alterations in muscle quality beyond simple atrophy. Our study lacks data on muscle strength, but decreased stiffness is associated to limb muscle weakness in healthy subjects and patients with chronic muscle diseases [15, 23, 24]. In critically ill patients, we demonstrated the association between decreased stiffness and loss of diaphragm force production [16]. Contrarily, muscle fibrosis that occurs in late ICU‐acquired weakness (32 [18–46] days of ICU stay) is associated with increased stiffness as reported in a pilot study with a one‐point late measurement [25].

To better understand the mechanisms behind the effects of high‐calorie intake at the acute phase of critical illness on skeletal muscle, we conducted a 72‐h mechanically ventilated piglet model with high‐calorie intake started at Day 0. Similarly to ICU patients, we found a decrease in ultrasound measurements of muscle mass and stiffness (Figure 3A,B). To make a parallel with simple muscular biopsies parameters, we evaluated at similar time points fibres CSA and intramuscular lipid accumulation, which, respectively, account for muscle mass and stiffness [16]. Muscle fibres CSA decreased by 10% between D0 and D3 (2257 μm^2^ vs. 2024 μm^2^, *p* = 0.64; Figure 3C). This difference was not statistically significant probably due to our small sample. Claassen et al. reported in 10 non‐septic ICU patients a nonstatistically significant 17% decrease in muscle fibre during the first week of critical illness, which was explained by the low number of patients included, even with a significantly lower muscle strength force over time [26]. The relatively low difference in fibre CSA may also be related with the healthy state of our model. Indeed, sepsis and high disease severity increase the risk of muscle dysfunction and atrophy [3, 4]. Intramuscular lipid accumulation increased significantly after 3 days of IMV (Figure 3D,E). Finally, both histological atrophy (Figure 3G,H) and increased intramuscular lipid content (Figure 3I,J) were significantly associated with the decrease in stiffness by more than 10%. This result is in line with our previous work on diaphragm [16]. High intramuscular lipid was also found correlated with lower muscle stiffness in other clinical situations like patients with metabolic syndrome [27].

Then, we investigated the modification of key expression factors involved in muscle homeostasis between D0 and D3. Our preconceived hypothesis based on previous works is that limb muscle immobilization leads to a state of relative energetic oversupply that would trigger the mechanisms of muscle wasting in critically ill patients [16, 28]. A high‐calorie intake during the early phase of critical illness may worsen those metabolic disorders encompassing protein and lipid metabolism. Regarding protein metabolism and muscle mass homeostasis (Figure 4), we found a downregulation of IGF‐1, a key growth factor involved in muscle protein synthesis as previously reported in critically ill patients [4]. However the proteolytic activity of the proteasome system remained unchanged, indicating constant catabolic activity, suggesting a negative protein balance [2]. As a possible compensatory mechanism to counteract IGF‐1 downregulation, myostatin, a myokine that suppresses muscle growth, was downregulated. Autophagy was activated in the present study. Autophagy is activated in fasting, oxidative stress and denervation, resulting in significant muscle protein degradation [4, 7]. Thus, our model of 3‐day mechanically ventilated piglets receiving high‐calorie intake from Day 0 revealed signs of anabolic resistance at the level of limb muscle with downregulation of protein synthesis without activation of proteolysis but associated to autophagy activation. Cacciani et al. reported similar general transcriptional downregulation of myofibrillar proteins and activation of protein degradation pathways in non‐septic mechanically ventilated neuro‐ICU patients resulting in significant muscle fibre atrophy and loss in force generation capacity [7]. The remaining question is what is the specific role of a high‐calorie intake in these disorders. Banduseela et al. used a similar piglet model mimicking the basic ICU interventions (immobilization, sedation and IMV) but without nutritional support. Interestingly, they reported no atrophy despite a downregulation of protein synthesis and increased proteasome activity with upregulation of autophagy [29]. In their next study, they compared this basic ICU piglet model to a model with critically ill muscle‐damaging interventions including sepsis, steroids and neuromuscular blockade and still without nutritional support. They comparatively reported a significant decrease in protein synthesis with similar expression of proteolytic activity but with impaired autophagy [30]. Regarding lipid metabolism, we found a downregulation of muscle CPT1b, a transporter of fatty acids into mitochondria, which could be linked to a lipid accumulation near mitochondria (Figure 5A,B). Indeed, mouse models with muscle‐specific CPT1b knockouts experienced impaired mitochondrial fatty acid oxidation (FAO), creating a feedback loop that signals lipid shortage in muscle, prompting a rise in genes responsible for lipid mobilization [31]. In mechanically ventilated brain donor patients (57 ± 13 h) with diaphragm biopsies, Picard et al. reported that metabolic oversupply resulting from diaphragm inactivity is a potential instigator of mitochondrial dysfunction and oxidative stress, which result in intramuscular lipid accumulation associated with a downregulation of CPT1b. These changes were not found in Bb biopsies [28]. Similarly in the present study, we observed no morphological changes in mitochondrial ultrastructure and similar Cox staining between D0 and D3 suggesting a normal mitochondrial capacity to produce energy, supported by stable expression levels of PGC‐1α, a key regulator of mitochondrial biogenesis. The absence of impaired mitochondrial function in our study is probably related to the healthy state of the piglet model, in comparison to animal sepsis models and critically ill [3, 4, 9, 19]. Indeed, we found no correlation between oxidative metabolic capacity and the amount of intramuscular lipids (Figure 5F), paralleling observations from a secondary analysis of the MUSCLE study where inflammation in the critical illness was associated to reduced mitochondrial biogenesis and impaired lipid metabolism with lipid delivery observed as bioenergetically inert [19]. Together, these findings suggest that early and high‐calorie nutritional support may understate the decrease in protein synthesis and the activation of protein degradation pathways, but these pathways do not appear to represent the primary cellular mechanism underlying muscle atrophy and weakness in the acute phase of ICU stay. Excessive autophagy may play a role in limb muscle atrophy related to excessive nutrition; mitochondrial dysfunction is to be linked to inflammation of critical illness rather than the metabolic oversupply resulting from high nutritional intake to muscle cells which are unable to use it for muscle building.

Previously, early high‐calorie nutrition with high‐protein intake was proposed to prevent muscle weakness and atrophy associated to critical illness‐induced catabolism, but recent data showed lack of benefit or even harm associated to these strategies [2, 9]. The EFFORT and PRECISE randomized trials showed that high‐protein intake during the early phase does not enhance outcomes and can negatively affect patient quality of life [32, 33]. Among hypothesis to explain this negative effect, critically ill patients showed impaired capacity of to use ingested proteins [34]. When critically ill muscle‐damaging interventions like sepsis occur, ketogenic nutrition leads to a state of muscular bioenergetic failure, which exacerbates muscle weakness [35]. Interestingly, a pilot study reported that protein administration after 10 days of critical illness significantly improved nutritional status, basal metabolism and muscle strength in ICU patients [36]. We can hypothesize that high‐calorie and protein intakes during the early phase of ICU stay lead or exacerbate impairments of muscle protein homeostasis reflecting anabolism resistance, a changing phenomenon over time in critically ill patients according to the stress response, the inflammatory and endocrine alterations and to the relative immobilization of the patients [37]. Similarly to the anabolic resistance for protein intake, concerns might be raised about the physiological ability of ICU patients to effectively metabolize lipid at the acute phase of critical illness. The intramuscular lipid accumulation is present in both diaphragm and limb muscle of immobilized mechanically ventilated patients and seems to be in part related to the downregulation of CPT1b. Puthucheary et al. made similar hypothesis, when reporting that the quantity of lipids delivered by enteral nutrition was unrelated to the amount of energy in the muscle (ATP content) and to the changes in quantity of skeletal muscle [19].

All put together, these results indicate that an early metabolic oversupply (imbalance between high intake of calories and metabolic substrates into muscle relative to low energetic consumption because of immobilization and sedation) leads to an energy crisis in skeletal muscle. We describe the complexity of metabolic responses to nutrition with anabolic resistance (defect in protein synthesis despite sufficient protein intake) leading to muscle atrophy, autophagy activation and muscle cells inability to use lipids as a fuel, resulting in its intramuscular accumulation. That may partly explain the conflict between observational and interventional studies about clinical benefit or harm from early targeted nutrition.

Our study presents limits that should be discussed. First, the absence of serial muscle biopsies in patients restricts direct correlations between ultrasound findings and muscle pathology. We therefore could only describe the association between ultrasound measurements and biopsy findings in the piglet model. Nevertheless, our findings correlate with recent data from the clinical and fundamental literature. Second, we selected to evaluate Bb over quadriceps to mitigate variability concerns for mass and quality measurements [14, 21]. We acknowledge that this choice may not fully represent the common patterns observed in critically ill patients. Anyway, upper and lower limb muscles wasted similarly in critically ill patients [38, 39]. Third, while ultrasound assessments provided valuable insights into muscle mass and quality, reliance on these measures does not analyse directly muscle strength and endurance, which are critical to understanding nutritional impacts on recovery. We unfortunately did not record functional assessments such as the Medical Research Council score or handgrip strength. Anyway, Bb atrophy is associated to muscle weakness measured and decreased stiffness was reported to be associated with muscle weakness in diaphragm and limb muscles [16, 22, 24]. An ongoing study is dedicated to explore the association between SWE measurement, early detection of muscle weakness (ultrasound and functional assessment) and their relationship with IMV weaning in critically ill patients (NCT04550143). Fourth, we did not perform standardized quantitative echo intensity analysis of B‐mode images in patients. Because ultrasound acquisition parameters (gain, depth, dynamic range and time‐gain compensation) were not prospectively fixed and raw DICOM data were not systematically stored, a reliable post hoc greyscale analysis could not be conducted. As a result, we could not directly quantify intramuscular fat infiltration in the clinical cohort and had to rely on the piglet model to link ultrasound changes to histological alterations. Another limitation is that the experimental model used healthy piglets exposed to sedation, immobilization, IMV and high‐calorie nutrition, but not to sepsis or systemic inflammation. In contrast, most patients in our clinical cohort had sepsis. Sepsis models typically show more profound mitochondrial dysfunction, oxidative stress and inflammation‐driven catabolism, which may further modify protein and lipid metabolism. Therefore, our experimental findings likely represent the effects of nutritional oversupply in the absence of severe systemic inflammation and may underestimate the complexity of metabolic derangements in septic ICU patients. Finally, the study was conducted in a single centre, which may limit the generalizability of our results due to variations in practice patterns, patient characteristics and nutritional protocols across different institutions. However, our extensive data collection from 102 patients, with over 600 ultrasound examinations overall ICU stay whereas other longitudinal studies are typically capping follow‐up at Day 7 or Day 10, contributes to the validity and robustness of our conclusions.

In summary, the present study emphasizes that high‐calorie intake may disrupt metabolic homeostasis and impair muscle function, which worsens ICU outcomes. The piglet model reinforces these observations, revealing morphological and metabolic alterations associated with the metabolic oversupply due to early excessive calorie intake. Future research should focus on integrating real‐time muscle assessment to optimize targeted nutrition strategies that consider muscle metabolism to enhance patient outcomes in critical care settings.

## Funding

This study received funding from an INSERM‐transfer research grant, the Medical School of Montpellier (Yassir Aarab, master research grant), and the RegenHab FHU (Aurelien Flatres, University Hospital of Montpellier, PhD grant).

## Ethics Statement

Institutional ethical approval was obtained (2017‐CLER‐MTP‐09‐16), and the study was registered (ClinicalTrial NCT03550222). An additional ethical approval was obtained (2025‐11‐368) for nutritional and peripheral muscle data. Because the present study was part of an image databank storage, consent was waived according to French law. Next of kin were informed of the study, as were patients as soon as their neurologic status was deemed adequate. Because the study was purely observational, consent was waived.

## Conflicts of Interest

Pr. Jaber reports receiving consulting fees from Drager, Medtronic, Mindray, Fresenius, Baxter and Fisher & Paykel. No potential conflicts of interest relevant to this article were reported for the other authors.

## Supporting information


**Figure S1:** Illustration showing the placement of the ultrasound probe (A) and a biceps brachii elastogram (B).
**Figure S2:** Illustrations showing animal preparation, images acquisition and muscle biopsies sampling. (A) Presentation of the animal laboratory with two sedated piglets and mechanically ventilated at the same time on Day 0 before the start of data collection. (B) Ultrasound images acquisition on the quadriceps of the right paw at Day 0. (C) Illustration showing a quadriceps muscle biopsy performed at Day 3 on the marked area of previous images acquisition. (D) Muscle biopsy sample harvested from the quadriceps muscle of the right paw.
**Figure S3:** Mediation analysis. Relationships between Exposure = Early calorie intake, Mediator = Biceps brachii thickness or stiffness trajectories and Outcomes = Ventilatory‐free days at Day 28, ICU stay. Total effect (d): the difference in Outcome among patients with different Exposures. Direct effect (c): a change in Outcome attributable to Exposure when the Mediator is fixed (ADE, average direct effect). Indirect effect (ab): a change in Ouctome that is attributable to Mediator while adjusting for the Exposure (ACME, average causal mediation effect). Adjusted on confounders (variables that are associated with the exposure and the mediator or the outcome) = age, gender, SOFA score, SAPSII score, sepsis, septic shock, use of NMBA, use of corticosteroids and need for dialysis.
**Figure S4:** Relationship between thickness and stiffness over time in critically ill patients. Thickness and stiffness are, respectively, represented in abscissa and ordinate as absolute values (A) and as percentage of change from baseline (B). Each point represents 1 day of measurement for one patient. No correlation was found between biceps brachii thickness and stiffness as absolute values, but percentage of variation from baseline (percentage) were significatively correlated.
**Figure S5:** Mediation analysis. Mediation of the association between Early high‐calorie intake and Ventilatory‐free days at Day 28 by biceps brachii decreased thickness trajectory. Adjusted on confounders = age, gender, SOFA score, SAPSII score, sepsis, septic shock, use of NMBA, use of corticosteroids and need for dialysis.
**Figure S6:** Mediation analysis. Mediation of the association between Early high‐calorie intake and Ventilatory‐free days at Day 28 by biceps brachii decreased stiffness trajectory. Adjusted on confounders = age, gender, SOFA score, SAPSII score, sepsis, septic shock, use of NMBA, use of corticosteroids and need for dialysis.
**Figure S7:** Mediation analysis. Mediation of the association between Early high‐calorie intake and ICU stay by Biceps brachii decreased thickness trajectory. Adjusted on confounders = age, gender, SOFA score, SAPSII score, sepsis, septic shock, use of NMBA, use of corticosteroids and need for dialysis.
**Figure S8:** Mediation analysis. Mediation of the association between Early high‐calorie intake and ICU stay at Day 28 by biceps brachii decreased stiffness trajectory. Adjusted on confounders = age, gender, SOFA score, SAPSII score, sepsis, septic shock, use of NMBA, use of corticosteroids and need for dialysis.
**Figure S9:** Illustration showing biceps brachii thickness (A and B) and stiffness (C and D) measurements for all patients. Absolute value (A and C) and changes over time expressed as percentage from baseline (B and D) of all biceps brachii recordings.
**Table S1:** Observed changes from baseline to the final measurement at the end of the first week for the three key variable: Bb thickness, Bb stiffness and daily calorie intake. Overall study population and comparison between calorie groups. All data are presented as mean (±standard deviation), or median [interquartile] for continuous variables, as appropriate. Results are presented for the total study population (102 patients).
**Table S2:** Univariate analysis of outcomes according to changes in Bb thickness and Bb stiffness over time. *Definition of abbreviations*: ICU = intensive care unit; VFD28 = Ventilator‐free day at Day 28 All data are presented as either *n* (%) for categorical variables; or median [Interquartile] for continuous variables, as appropriate. Results are presented for the total study population (102 patients). *: Durations are expressed in days.
**Table S3:** Factors associated with outcomes. Adjusted multivariate analysis. Definition of abbreviations: CI = Confidence interval; ICU = Intensive Care Unit. Adjusted on age, gender, SOFA score, SAPSII score, sepsis, septic shock, use of NMBA, use of corticosteroids and need for dialysis. A linear regression analyses were performed for VFD28 and ICU stay. A Cox regression analysis was used for ICU mortality.
**Table S4:** Effects of early calorie intake on outcomes—Adjusted mediation analysis modelling with ‘early high‐intake’ as exposure ‘decreased trajectory groups’ for thickness and stiffness as mediators and ‘VFD 28’ and ‘ICU stay’ as outcomes. *Definition of abbreviations*: Bb = Biceps Brachii; ICU = intensive care unit; OR = Odd Ratio; VFD28 = Ventilatory‐free Days at Day 28. Adjusted on age, gender, SOFA score, SAPSII score, sepsis, septic shock, use of NMBA, use of corticosteroids and need for dialysis. This mediation analysis is employed to elucidate the relationships among the exposure variable ‘**early high‐intake**’, a mediator variable ‘**decreased trajectory groups**’ **for thickness and stiffness** and an outcome variable ‘**VFD 28**’ and ‘**ICU stay**’. First, an adjusted linear regression analysis and an adjusted logistic regression analysis were used to, respectively, examine the relationship between exposure and outcome validating their association, and between exposure and mediator, establishing whether the mediator functions as a significant conduit. Once these relationships validated, a second linear regression analysis was then performed, to explore the connection between the mediator and the outcome while controlling for the exposure, and to examine the relationship between exposure and outcome when incorporating the mediator as a predictor. Finally, we evaluated the total, direct and indirect (mediated) effects of the exposure on the outcome and calculated the proportion of the mediated effect employing a 5000‐simulations bootstrapping method.

## Data Availability

The data that support the findings of this study are available on request from the corresponding author. The data are not publicly available due to privacy or ethical restrictions.
