# Supplementary material for: Changes in Muscle Mass and Quality Mediate the Effect of High‐Calorie Intake on Clinical Outcomes
Source: J Cachexia Sarcopenia Muscle. 2026 Aug 12;17(4):e70355. doi: 10.1002/jcsm.70355 (PMC13469338; doi:10.1002/jcsm.70355)
Supplement: Supplementary file 1 [file JCSM-17-e70355-s001.docx]

**SUPPLEMENTAL FIGURES**

**Figure E1: Illustration showing the placement of the ultrasound probe (A), and a biceps brachii elastogram (B).**

**igure E2: Illustrations showing animal preparation, images acquisition and muscle biopsies sampling**

(A) Presentation of the animal laboratory with two sedated piglets and mechanically ventilated at the same time on day 0 before the start of data collection
(B) Ultrasound images acquisition on the quadriceps of the right paw at day 0
(C) Illustration showing a quadriceps muscle biopsy performed at day 3 on the marked area of previous images acquisition
(D) Muscle biopsy sample harvested from the quadriceps muscle of the right paw

**Figure E3: Mediation analysis.**

Relationships between Exposure = Early calorie intake, Mediator = Biceps brachii thickness or stiffness trajectories and Outcomes = Ventilatory-free days at day 28, ICU stay.

. Total effect (d): the difference in Outcome among patients with different Exposures.

. Direct effect (c): a change in Outcome attributable to Exposure when the Mediator is fixed (ADE, average direct effect).

. Indirect effect (ab): a change in Ouctome that is attributable to Mediator while adjusting for the Exposure (ACME, average causal mediation effect).

Adjusted on confounders (variables that are associated with the exposure and the mediator or the outcome) = age, gender, SOFA score, SAPSII score, sepsis, septic shock, use of NMBA, use of corticosteroids and need for dialysis.

**Figure E4: Relationship between thickness and stiffness over time in critically ill patients**

Thickness and stiffness are respectively represented in abscissa and ordinate as absolute values **(A)** and as percentage of change from baseline **(B)**. Each point represents one day of measurement for one patient.

No correlation was found between biceps brachii thickness and stiffness as absolute values, but percentage of variation from baseline (percentage) were significatively correlated.

**Figure E5: Mediation analysis. Mediation of the association between Early high-calorie intake and Ventilatory-free days at day 28 by Biceps brachii decreased thickness trajectory .** Adjusted on confounders = age, gender, SOFA score, SAPSII score, sepsis, septic shock, use of NMBA, use of corticosteroids and need for dialysis

**Figure E6: Mediation analysis. Mediation of the association between Early high-calorie intake and Ventilatory-free days at day 28 by Biceps brachii decreased stiffness trajectory**

Adjusted on confounders = age, gender, SOFA score, SAPSII score, sepsis, septic shock, use of NMBA, use of corticosteroids and need for dialysis

**Figure E7: Mediation analysis. Mediation of the association between Early high-calorie intake and ICU stay by Biceps brachii decreased thickness trajectory**

Adjusted on confounders = age, gender, SOFA score, SAPSII score, sepsis, septic shock, use of NMBA, use of corticosteroids and need for dialysis

**Figure E8: Mediation analysis. Mediation of the association between Early high-calorie intake and ICU stay at day 28 by Biceps brachii decreased stiffness trajectory**

Adjusted on confounders = age, gender, SOFA score, SAPSII score, sepsis, septic shock, use of NMBA, use of corticosteroids and need for dialysis

**Figure E9: Illustration showing biceps brachii thickness (A and B) and stiffness (C and D) measurements for all patients. Absolute value (A and C) and changes over time expressed as percentage from baseline (B and D) of all biceps brachii recordings.**

**SUPPLEMENTAL TABLES**

| Variables | Study population  (n=102) | Daily caloric intake during the first week | | |
| --- | --- | --- | --- | --- |
|  |  | High-intake  (n=37) | Standard-intake  (n=65) | P value |
| Bb thickness on admission (cm) | 2.2 (±0.4) | 2.2 (±0.4) | 2.2 (±0.6) | 0.57 |
| Bb thickness at day 7 (cm) | 2.0 (±0.4) | 1.9 (±0.3) | 2.1 (±0.4) | 0.02 |
| Bb stiffness on admission (cm) | 25.2 (± 7.0) | 25.9 (±7.4) | 24.0 (±6.2) | 0.18 |
| Bb stiffness on at day 7 (kPa) | 18.6 (± 7.2) | 11.6 (±6.7) | 22.7 (±7.3) | <0.01 |
| Daily calorie intake at day 1 (kcal/kg) | 5.6 [1.1-13.2] | 7.6 [2.8-15.2] | 4.5 [1.5-12.3] | 0.65 |
| Daily calorie intake at day 7 (kcal/kg) | 23.4 [13.0-27.0] | 28.6 [26.6-32.4] | 16.0 [7.1-21.9] | <0.01 |

**Table E1. Observed changes from baseline to the final measurement at the end of the first week for the three key variable: Bb thickness, Bb stiffness and daily calorie intake. Overall study population and comparison between calorie groups**

All data are presented as mean (± Standard Deviation), or median [Interquartile] for continuous variables, as appropriate.

Results are presented for the total study population (102 patients).

Regarding calorie intake, a threshold of 25 kcal/kg/day at day 7 was employed:

Patients in the “early standard-intake group” have a daily caloric intake lower than 25 kcal/kg/day at day 7.

Patients in the “early high-intake group” have a daily caloric intake hgher than 25 kcal/kg/day at day 7.

**Table E2. Univariate analysis of outcomes according to changes in Bb thickness and Bb stiffness over time.**

| Outcome | Study population  (n=102) | Biceps brachii thickness trajectory groups | | |
| --- | --- | --- | --- | --- |
|  |  | Decreased group  (n=21) | Stable group  (n=81) | P value |
| VFD28* | 21 [11-25] | 4.1 [1.0-12.5] | 21.7 [11.2-25.0] | <0.01 |
| ICU stay | 12 [7-19] | 28.7 [19.0-35.5] | 10.3 [6.7-14.3] | <0.01 |
| ICU mortality | 23 (23) | 5 (24) | 18 (22) | 0.88 |
| Outcome | Study population  (n=102) | Biceps brachii stiffness trajectory groups | | |
|  |  | Decreased group  (n=29) | Stable group  (n=73) | P value |
| VFD28 | 21 [11-25] | 4.4 [0.0-17.8] | 21.7 [11.0-25.2] | <0.01 |
| ICU stay | 12 [7-19] | 17.2 [12.2-28.0] | 9.5 [6.3-17.0] | <0.01 |
| ICU mortality | 23 (23) | 10 (35) | 13 (18) | 0.07 |

*Definition of abbreviations*: ICU = intensive care unit; VFD28 = Ventilator-free day at day 28

All data are presented as either n (%) for categorical variables; or median [Interquartile] for continuous variables, as appropriate.

Results are presented for the total study population (102 patients).

^*^: Durations are expressed in days.

**Table E3. Factors associated with outcomes. Adjusted multivariate analysis**

| Risk factors |  | **Ventilator free days at day 28** | | |
| --- | --- | --- | --- | --- |
|  | (/ reference) | Linear regression coefficient | 95% CI | P Value |
| Thickness trajectory group | Stable/ Decrease | 8.84 | 4.63 - 13.05 | <0.01 |
| Calorie group | High intake/ Standard intake | -8.28 | -11.76 - -4.81 | <0.01 |
| SAPS II score |  | -0.13 | -0.25 - -0.02 | 0.03 |
| Risk factors |  | **ICU stay** | | |
|  | (/ reference) | Linear regression coefficient | 95% CI | P Value |
| Thickness trajectory group | Stable/ Decrease | -16.70 | -22.27 – -11.13 | <0.01 |
| Risk factors |  | **ICU mortality** | | |
|  | (/ reference) | Hazard ratio | 95% CI | P Value |
| Age |  | 1.04 | 1.00 – 1.08 | 0.04 |
| Thickness trajectory group | Stable/ Decrease | 0.20 | 0.05 - 0.78 | 0.02 |

Definition of abbreviations: CI = Confidence interval; ICU = Intensive Care Unit.

Adjusted on age, gender, SOFA score, SAPSII score, sepsis, septic shock, use of NMBA, use of corticosteroids and need for dialysis.

A linear regression analyses were performed for VFD28 and ICU stay.

A Cox regression analysis was used for ICU mortality.

**Table E4. Effects of early calorie intake on outcomes – Adjusted mediation analysis modelling with “early high-intake” as exposure “decreased trajectory groups” for thickness and stiffness as mediators and “VFD 28” and “ICU stay” as outcomes**

| **Outcome** | **Mediator** | **Effect of high-intake on outcome using adjusted linear regression (Estimator [95% CI];**  **p-value)** | **Effect of high-intake on mediator using an adjusted logistic regression (OR [95% CI]; p-value)** | **Effect of mediator on outcome when adjusted on high-intake (Estimator [95% CI]; p-value)** | **Effect of high-intake on outcome when adjusted on mediator (Estimator [95% CI]; p-value)** | **Total effect of high- intake on outcome (Estimator [95% CI];**  **p-value)** | **Direct effect of high- intake on outcome (Estimator [95% CI];**  **p-value)** | **Inirect (mediated) effect of calorie intake on outcome (Estimator [95% CI]; p-value)** | **Proportion mediated**  **(% [95% CI]; p-value)** |
| --- | --- | --- | --- | --- | --- | --- | --- | --- | --- |
| VFD 28 | Bb decreased thickness trajectory | -8.9  [-15.8- -2.0]; p=0.01 | 6.25  [1.28-33.3]; p=0.02 | -9.6  [-13.8- -5.5]; p<0.01 | -5.0  [-1.5-11.5]; p=0.13 | -8.7  [-14.7- -1.7]; p=0.01 | -5.0  [-10.1- -0.8]; p=0.08 | -3.7  [-7.8- -0.2]; p=0.04 | 42%  [4-99];  p=0.04 |
| VFD 28 | Bb decreased stiffness trajectory | -8.9  [-15.8- -2.0]; p=0.01 | 10.0  [2.9-33.3]; p<0.01 | -6.0  [-10.6- -1.5]; p<0.01 | -6.5  [-11.7- -1.2]; p=0.02 | -8.9  [-13.7- -4.7]; p<0.01 | -6.5  [-11.3- -1.0]; p=0.02 | -2.5  [-0.4- -6.6]; p=0.01 | 28%  [4-84];  p=0.04 |
| ICU stay | Bb decreased thickness trajectory | 8.4  [2.7-14.0]; p<0.01 | 5.3  [1.6-16.7]; p<0.01 | 15.7  [9.4-22]; p<0.01 | 4.8  [0.2-9.7]; p=0.07 | 7.7  [1.8-16.5]; p<0.01 | 4.8  [0.2-9.7]; p=0.04 | 2.9  [0.2-9.1]; p=0.03 | 38%  [4-90%]; p=0.03 |
| ICU stay | Bb decreased stiffness trajectory | 8.4  [2.7-14.0]; p<0.01 | 3.3  [1.2-9.1]; p=0.02 | 5.1  [1.3-11.4]; p=0.12 | 7.3  [1.6-13.1]; p=0.02 | 8.6  [1.9-16.0]; p<0.01 | 7.3  [0.9-14.4]; p=0.02 | 1.2  [-0.4-3.7]; p=0.19 | 14%  [0-60%]; p=0.20 |

*Definition of abbreviations*: Bb = Biceps Brachii; ICU = intensive care unit; OR = Odd Ratio; VFD28 = Ventilatory-free Days at day 28.

Adjusted on age, gender, SOFA score, SAPSII score, sepsis, septic shock, use of NMBA, use of corticosteroids and need for dialysis.

This mediation analysis is employed to elucidate the relationships among the exposure variable **“early high-intake”,** a mediator variable **“decreased trajectory groups” for thickness and stiffness**, and an outcome variable **“VFD 28”** and **“ICU stay”**.

First, an adjusted linear regression analysis and an adjusted logistic regression analysis were used to respectively examine the relationship between exposure and outcome validating their association, and between exposure and mediator, establishing whether the mediator functions as a significant conduit.

Once these relationships validated, a second linear regression analysis was then performed,to explore the connection between the mediator and the outcome while controlling for the exposure, and to examine the relationship between exposure and outcome when incorporating the mediator as a predictor.

Finally, we evaluated the total, direct and indirect (mediated) effects of the exposure on the outcome and calculated the proportion of the mediated effect employing a 5000-simulations bootstrapping method.
